# Managing multiple chronic conditions in the community: a Canadian qualitative study of the experiences of older adults, family caregivers and healthcare providers

**DOI:** 10.1186/s12877-017-0431-6

**Published:** 2017-01-31

**Authors:** Jenny Ploeg, Nancy Matthew-Maich, Kimberly Fraser, Sinéad Dufour, Carrie McAiney, Sharon Kaasalainen, Maureen Markle-Reid, Ross Upshur, Laura Cleghorn, Anna Emili

**Affiliations:** 1 Aging, Community and Health Research Unit, School of Nursing, Faculty of Health Sciences, McMaster University, 1280 Main Street West, HSc3N25C, Hamilton, ON L8S 4K1 Canada; 2 Department of Health, Aging and Society, McMaster University, 1280 Main Street West, HSc3N25C, Hamilton, ON L8S 4K1 Canada; 3 Health Science Research and Innovation, School of Nursing, Mohawk College of Applied Arts and Technology, 1400 Main Street West, IAHS - 354, Hamilton, ON L8S 1C7 Canada; 4 Faculty of Nursing, University of Alberta, 5-185 Edmonton Clinic Health Academy, Edmonton, AB T6G 1C9 Canada; 5 School of Rehabilitation Science, Faculty of Health Sciences, McMaster University, 1400 Main Street West, IAHS Rm 403, Hamilton, ON L8S 4K1 Canada; 6 Department of Psychiatry & Behavioural Neurosciences, Faculty of Health Sciences, McMaster University, St. Joseph’s Healthcare Hamilton, West 5th Campus, 100 West 5th Street, Room G102, Hamilton, ON L8N 3K7 Canada; 7 School of Nursing, Faculty of Health Sciences, McMaster University, 1280 Main Street West, Hamilton, ON L8S 4K1 Canada; 8 Aging, Chronic Disease and Health Promotion Interventions, 1280 Main Street West, HSc3N25B, Hamilton, ON L8S 4K1 Canada; 9 Clinical Epidemiology and Biostatistics, Faculty of Health Sciences, McMaster University, 1280 Main Street West, HSc3N25B, Hamilton, ON L8S 4K1 Canada; 10 Department of Family and Community Medicine and Dalla Lana School of Public Health, University of Toronto, M.33 1 Bridgepoint Drive, Toronto, ON M4M 2B5 Canada; 11 Bridgepoint Collaboratory for Research and Innovation, Sinai Health System, AM.33 1 Bridgepoint Drive, Toronto, ON M4M 2B5 Canada; 12 School of Nursing and Department of Family Medicine, Health TAPESTRY, McMaster University, 1280 Main Street West, David Braley Health Science Centre, 5th Floor, Hamilton, ON L9S 4K1 Canada; 13 McMaster University, Main West Medical Group, 1685 Main Street West, Hamilton, ON L8S 1G5 Canada

**Keywords:** Multimorbidity, Older persons, Caregivers, Healthcare providers, Primary care, Home care, Qualitative research

## Abstract

**Background:**

The prevalence of multiple chronic conditions (MCC) among older persons is increasing worldwide and is associated with poor health status and high rates of healthcare utilization and costs. Current health and social services are not addressing the complex needs of this group or their family caregivers. A better understanding of the experience of MCC from multiple perspectives is needed to improve the approach to care for this vulnerable group. However, the experience of MCC has not been explored with a broad sample of community-living older adults, family caregivers and healthcare providers. The purpose of this study was to explore the experience of managing MCC in the community from the perspectives of older adults with MCC, family caregivers and healthcare providers working in a variety of settings.

**Methods:**

Using Thorne’s interpretive description approach, semi-structured interviews (*n* = 130) were conducted in two Canadian provinces with 41 community-living older adults (aged 65 years and older) with three or more chronic conditions, 47 family caregivers (aged 18 years and older), and 42 healthcare providers working in various community settings. Healthcare providers represented various disciplines and settings. Interview transcripts were analyzed using Thorne’s interpretive description approach.

**Results:**

Participants described the experience of managing MCC as: (a) overwhelming, draining and complicated, (b) organizing pills and appointments, (c) being split into pieces, (d) doing what the doctor says, (e) relying on family and friends, and (f) having difficulty getting outside help. These themes resonated with the emotional impact of MCC for all three groups of participants and the heavy reliance on family caregivers to support care in the home.

**Conclusions:**

The experience of managing MCC in the community was one of high complexity, where there was a large gap between the needs of older adults and caregivers and the ability of health and social care systems to meet those needs. Healthcare for MCC was experienced as piecemeal and fragmented with little focus on the person and family as a whole. These findings provide a foundation for the design of care processes to more optimally address the needs-service gap that is integral to the experience of managing MCC.

## Background

The prevalence of multiple chronic conditions (MCC), defined as having two or more chronic conditions, has increased worldwide over the past two decades [1, 2]. This trend is even more pronounced in the older adult population, aged 65 and older, where the incidence of MCC, also called multimorbidity, markedly increases with age [3, 4]. Individuals aged 65 years and older with two or more chronic conditions represent 60.8% of noninstitutionalized older adults in the United States [1]. Older persons with MCC report poorer health status and have higher rates of healthcare utilization and costs compared to individuals with no or fewer conditions; they take six or more prescription medications, and are at high risk for adverse events such as death, hospitalization, and falls [5–7].

Effective healthcare for older persons with MCC is a global challenge and increasing priority. The U.S. Agency for Healthcare Research and Quality MCC Research Network (AHRQ MCCRN) has invested significant research funding to improve understanding in this area. An important product of this work was the development of a conceptual model of the role of complexity in the care of persons with MCC [8]. In this model, complexity is defined as the gap between an individual’s needs and the capacity of healthcare services to meet those needs [8]. This ecological model takes a holistic view of complexity, recognizing the influence of social support (e.g., family and other sources) as well as contextual factors (e.g., economic, social and physical) on the interaction between MCC-related needs and services.

A recent review of interventions for individuals with multimorbidity in community and primary care settings demonstrated variability regarding effectiveness [9]. Of the 18 randomized controlled trials included in the review, nine were focused on multimorbidity in older persons and nine were focused on defined conditions such as depression, diabetes and cardiovascular disease. In 12 studies the main intervention involved a change to the way care was delivered, usually through enhanced multidisciplinary team work or case management. The authors found no clear positive improvements in clinical outcomes, patient-related health behaviours, health professional behaviours, and health service use or costs, and concluded that it is difficult to improve outcomes for persons with MCC. Clearly there is a need to refine, refocus and improve the delivery of health and social services for older adults with MCC [10]. A vital starting point for designing improved services involves a sound understanding of the experiences and challenges of managing MCC from the perspectives of not only older adults with MCC, but also family caregivers (hereafter referred to as caregivers) and healthcare providers (referred to as providers).

A number of qualitative studies have explored the experiences of older adults with MCC. These studies have examined specific topics such as symptom burden [11], coping [12], health care practices [13], care planning [14] and the care processes desired by older adults [15]. Few studies have examined the experiences of informal caregivers of older persons with MCC, who provide 80–90% of the care at home for these individuals [16–18]. In contrast, there have been numerous studies of the experiences of healthcare providers of older persons with MCC. Most of these studies have explored the challenges experienced by general practitioners [19–21].

Only one study, reported in two publications, examined the combined experience of MCC from perspectives of patients, family caregivers and providers [16, 18]. The first paper explored the alignment of care goals among 28 older adults with MCC, their family caregivers and four family physicians at one primary care practice clinic [18]. They determined that common goals included the maintenance of functional independence and symptom management, and that alignment of care goals across the triad was present when the client had a stable health condition. However, in situations of declining client health, there was less clarity regarding future steps and more divergence in care goals [18]. The second paper reported care challenges experienced by the three groups while managing multimorbidity [16]. Similarities in themes were found regarding poor communication, lack of care coordination and long wait times for both appointments and feedback; discrepancies in themes were found in areas such as access to care and treatment nonadherence.

The studies by Gill et al. and Kuluski et al. [16, 18] address the need for in-depth understanding of the experiences of all three groups, older adults, caregivers and providers, to develop and provide more comprehensive, optimal and consistent approaches to care that address the complexities of MCC. Study participants represented only one care setting, a family practice clinic, in an affluent urban neighbourhood, which limits the applicability of results to other care settings such as home care and more demographically diverse populations. The only providers included in these studies were primary care physicians. Research is needed on the experiences of MCC from the perspectives of a broader sample of older adults, caregivers and healthcare providers to inform the design of approaches to MCC that could improve outcomes for older adults and their caregivers. The purpose of this study was to explore the experience of managing MCC in the community from the perspectives of older adults, caregivers and providers. These study findings could yield important information to help develop innovative interventions to improve the ability of older adults, caregivers and providers to manage MCC. The research question was: What is the experience of managing MCC in the community from the perspectives of older adults, family caregivers and healthcare providers?

## Methods

### Design

We used a qualitative interpretive description (ID) approach to explore the experience of managing MCC [22]. This approach is suited to studies that seek to provide an in-depth understanding of a concept while generating results that will inform and guide clinical practice [22]. ID produces a thematic description of a phenomenon, while at the same time, honoring the complexity of the whole picture, acknowledging that multiple interpretations exist in a given context. In this way, ID is a suitable approach to guide inquiry into the experience of managing MCC from the multiple perspectives of older adults, caregivers and providers.

### Ethics

Ethics approval for this study was granted by the Hamilton Integrated Research Ethics Board, (#13-411), in Hamilton, Ontario and University of Alberta, Health Research Ethics Board, (#39559), in Edmonton, Alberta. A research coordinator or trained research assistant obtained written informed consent and each participant received a copy of the signed form.

### Sample

We used purposive sampling, consistent with the ID approach [22]. To ensure representative credibility, there was a broad sampling strategy involving two Canadian provinces (Alberta and Ontario) and three groups of participants: older adults with MCC, informal caregivers of older adults with MCC and healthcare providers who provide care to older adults with MCC. Inclusion criteria for older adults were: 65 years of age or older, living in the community, able to converse in English and having three or more chronic conditions with at least one of diabetes, dementia or stroke. These conditions were selected because vascular diseases are the second most significant cause of death for Canadians and place a high burden on the healthcare system [6]. Caregivers (family and friends) were included if they were: 18 years of age or older, able to converse in English, and provided care to an older adult with three or more chronic conditions, one of which was diabetes, dementia or stroke. Healthcare providers were included if they provided care to community-living older adults with MCC aged 65 years and older.

### Recruitment

Participants were primarily recruited through partner sites including: home care organizations, primary care practices, health clinics and collaborating community support associations. For older adults and caregivers, partner site recruiters identified potential participants. A representative from the site, not associated with the care of the older adult, contacted the potential participant by phone or in-person. For those who consented to receive further information about the study, a research coordinator telephoned and confirmed eligibility. Healthcare providers were recruited through email invitations sent by site-specific recruiters to staff members and community advertisements. Interested healthcare providers contacted the research coordinator who sent study information and confirmed eligibility. A recruitment target of 20 per group per province was set to facilitate maximum variation in patient, caregiver and provider groups, chronic conditions, and ultimately, experience of MCC. Sampling continued until data saturation was reached.

### Data collection

We collected data from July 2013 to June 2014. Face-to-face semi-structured audio-taped interviews were carried out by a research coordinator or research assistant trained in conducting qualitative interviews. Following written consent, demographic questionnaires were completed. Interviews were digitally recorded, averaged one hour in length and followed an open-ended interview guide tailored to each participant group (See Tables 1, 2 and 3). In total, 130 interviews were completed, 41 with older persons, 47 with caregivers and 42 with providers; 67 of the interviews took place in Ontario (ON) and 63 in Alberta (AB). Interviews took an average of 60 min.

**Table 1:** Interview Guide for Older Adults with Multiple Chronic Conditions

Experiences in Managing Multiple Chronic Conditions
1. Tell me about your experiences in living with more than one chronic condition at a time.
2. What do you do to prevent your chronic conditions from getting worse or new ones from developing?
3. How do you make decisions about what chronic conditions or symptoms to manage first?
4. How do you manage taking medications for your chronic conditions?
Facilitators in Managing Multiple Chronic Conditions
5. What helps you to manage your chronic conditions (i.e. people, resources)?
Challenges in Managing Multiple Chronic Conditions
6. What are some of the challenges you face in living with more than one condition at a time?
7. What makes it difficult for you to manage more than one chronic condition at a time?
Family Caregivers (only ask if client has listed a caregiver on the Demographic questionnaire)
8. You listed that you have (a) family caregiver(s); can you tell me more about how your caregiver helps you manage your chronic conditions.
9. What makes it difficult for your family caregiver to support you?
10. Is there anything else you would like them to do for you? Explain.
Health and Social Services
11. What supports do you receive to help you live with more than one condition at a time?
12. Do these services help you to manage your conditions? Please explain.
13. Older adults living with more than one condition often have multiple healthcare providers and services. How is your care coordinated among these providers and services? How could your care be better coordinated?
Discussing Conditions and Making Treatment Decisions with Health Professionals
14. Have you talked with health professionals about managing more than one chronic condition at a time? Tell me about that.
15. How have you worked with the health professionals in making decisions about your care?
16. Can you give me an example of a situation where you did not get the help you needed related to managing more than one chronic condition?
Treatment and Care Preferences
17. What do you hope to achieve in your management of more than one chronic condition at a time?
18. In order to achieve these outcomes what types of help would you find most useful?
19. What technology supports do you use to manage your chronic conditions? Please share your experience with using these.
20. Is there anything else you would like to share about managing more than one chronic condition at a time?

**Table 2:** Interview Guide for Family Caregivers of Older Persons with Multiple Chronic Conditions

Experiences in Managing Multiple Chronic Conditions
1. Tell me about your experiences in providing care to your family member who is managing more than one condition at a time?
2. How do you make decisions about what chronic conditions or symptoms to manage first?
3. How do you help your family member manage taking medications for all their chronic conditions?
Facilitators in Managing Multiple Chronic Conditions
4. What helps you to manage your family member’s chronic conditions (i.e. people, resources)?
Challenges and Rewards in Managing Multiple Chronic Conditions
5. What are some of the challenges you face in providing care to your family member who has a number of chronic conditions?
6. What makes it difficult for you to support them?
7. What are some of the rewards in your caregiving role?
Health and Social Services
8. What supports does your family member receive to help them live with more than one condition at a time?
9. What supports do you receive in your caregiving role? Describe.
10. Older adults living with more than one condition often have multiple healthcare providers and services. How is your family member’s care coordinated among these providers and services? How could these services be better coordinated?
Discussing Conditions and Making Treatment Decisions with Health Professionals
11. Have you talked with health professionals about ways to help your family member manage more than condition at a time? Tell me about that.
12. How have you worked with the health professional in making decisions about your family member’s care?
13. Can you give me an example of a situation where you did not get the help you needed to support your family member in managing more than one condition at a time?
Treatment and Care Preferences
14. What do you hope to achieve for your family member in their management of more than one condition at a time?
15. In order to achieve these outcomes what types of help would you find most useful in supporting your family member?
16. What technology supports do you use to help manage your family member’s chronic conditions? Please share your experience with using these.
17. Is there anything else you would like to share about being a caregiver for an older person with multiple chronic conditions?

**Table 3:** Interview Guide for Healthcare Providers

Experiences in Helping Older Patients to Manage Multiple Chronic Conditions
1. Tell me about your experiences in providing care to older patients who are managing more than one condition at a time.
2. What do you do to help them manage multiple chronic conditions?
3. How do you help older patients prevent their conditions from getting worse or new ones from developing?
4. How do you make decisions about managing more than one chronic condition at a time?
5. How do you make decisions about managing multiple medications?
6. From your experience what are the most important needs of family caregivers of older patients managing more than one condition at a time?
7. How do you support family members of older patients who have multiple chronic conditions?
Facilitators in Managing Multiple Chronic Conditions
8. What helps you to manage older patient’s chronic conditions (i.e. people, resources)?
Challenges and Rewards in Managing Multiple Chronic Conditions
9. What is your greatest challenge in helping older patients manage a number of conditions at the same time?
10. What makes it difficult for you to provide care to this group of patients?
11. What are some rewards in helping older patients manage a number of conditions at the same time?
Health and Social Services
12. Do you refer older patients with multiple chronic conditions to any health and social services?
Making Treatment Decisions with Health Professionals
13. Older patients with multiple chronic conditions often require multiple services. What mechanisms are in place to foster collaboration and coordination of care among multiple providers, services, settings and sectors?
14. How do you normally make treatment decisions with older patients who have multiple chronic conditions?
Goals of Care
15. What are your goals when caring for older adults with multiple chronic conditions?
16. How do you develop goals for the care of older patients with multiple chronic conditions?
17. How do you evaluate the success of the strategies that you use with this population?
18. What technology supports do you use to help support older patients with multiple chronic conditions? Please share your experience with using these.
19. Is there anything else you would like to share about providing care to older persons who have multiple chronic conditions, and supporting their family members?

### Data analysis

Data analysis was concurrent with data collection [22]. Data from all interviews were transcribed verbatim and cleaned. Transcripts were read and reread to ensure understanding of the parts within the context of the whole [22]. A team of experienced qualitative researchers independently coded the data and the codes were corroborated during regular meetings within and across provinces. Following the ID approach, during reading, researchers would ask themselves critical questions to examine the transcripts such as “Why is this here?” and “What is happening here?” [22]. We used an inductive approach to data analysis, moving from specific observations to broader generalizations; the AHRQ MCCRN model did not drive the analysis, but was used as a perspective from which to discuss the results.

Next, codes were merged into fewer, more encompassing patterns through discussions between research team members and patterns were further refined into more interpretive themes. We recorded our cross-province team meetings and used the recorded data to assist with final theme development. Constant comparative analysis was used to investigate similarities and differences within and across participant groups. Key quotes that illuminated each theme were extrapolated from the data. For the first 66 interviews completed (approximately 20 of each older adults, caregivers and providers), short one page summaries were created to describe the overall experience of the participant. These summaries were referred to throughout the comparative analysis and identification of patterns to maintain awareness of each participant’s unique experiences with MCC. To avoid potential bias in our analysis, the research team constantly compared the patterns identified in the summaries and first half of the transcripts to those in the later transcripts and together discussed confirming or new emerging trends.

Consistent with Thorne’s suggestions for quality using the ID approach, we used the following strategies: (a) “representative credibility” was ensured by triangulating data sources among three groups of participants, and by using a large sample from two provinces; (b) “analytic logic” was demonstrated by checking accuracy of transcription, data collectors writing memos to record context of data-gathering, keeping an audit trail, and using participants’ phrasing and verbatim accounts from the data; and (c) “interpretive authority” was maintained through iterative review of the transcripts, writing summaries of transcripts and involving seven different researchers as analysts, each challenging and questioning the interpretations of the others with their unique perspectives [22].

## Results

### Characteristics of the sample

Demographic characteristics of the three groups were similar across provinces as were the identified themes in the data. Thus, we pooled data across provinces. Older adults (*n* = 41) were primarily male (56.1%), 65–74 years old (58.5%), and married/common law (68.3%). Most older adults were living with 3–5 chronic conditions (41.5%) (Table 4), with an overall mean of 6.3 conditions. Caregivers (*n* = 47) were primarily female (76.6%), 65–74 years old (36.2%), married/common law (87.2%) and most often the spouse/common law partner (68.1%) of the person they supported (Table 5). Healthcare providers (*n* = 42) were largely female (95.2%), educated at the Bachelor’s level (40.5%), and represented a variety of professional backgrounds, the most common being Registered Nurse (28.6%), Registered/Licensed Practical Nurse (16.7%) and Personal Support Worker/Healthcare Aide (14.3%) (Table 6). The majority had been practicing for over 20 years (40.5%) and currently practiced in a primary care (47.6%) or home care (45.2%) setting.

**Table 4:** Description of Older Adults (*n* = 41)

Category	*n* (%)
Sex	
Female	18 (43.9)
Male	23 (56.1)
Age	
65–74	24 (58.5)
75–84	10 (24.4)
85+	7 (17.1)
Marital Status	
Single/Never Married	2 (4.9)
Married/Common Law	28 (68.3)
Widowed	5 (12.2)
Divorced	6 (14.6)
Highest Education Achieved	
Grade 8 or Less	6 (14.6)*
Some High School	7 (17.1)
Graduated High School	9 (22.0)
Technical or Trade School	2 (4.9)
Some University/College	7 (17.1)
Graduated University/College	7 (17.1)
Graduate Degree	3 (7.3)
Household Income	
$19,999 or Less	4 (9.8)
$20,000 to $39,999	14 (34.1)
$40,000 to $59,999	10 (24.4)
$60,000 or greater	9 (22.0)
Missing	4 (9.8)
Multiple Chronic Conditions	
3–5	17 (41.5)
6–8	12(29.3)
9–12	11 (26.8)
13	1 (2.4)

*Because of rounding, at times, percentages add up to 99.9–100.1

**Table 5:** Description of Family Caregivers (*n* = 47)

Category	*n* (%)
Sex	
Female	36 (76.6)
Male	11 (23.4)
Age	
18–44	4 (8.5)
45–64	14 (29.8)
65–74	17 (36.2)
75+	12 (25.5)
Marital Status	
Single/Never Married	4 (8.5)
Married/Common Law	41 (87.2)
Divorced Separated	2 (4.3)
Highest Education Achieved	
Some High School	7 (14.9)*
Graduated High School	14 (29.8)
Technical or Trade School	6 (12.8)
Some University/College	3 (6.4)
Graduated University/College	14 (29.8)
Graduate Degree	3 (6.4)
Relationship to Older Person Supported	
Spouse/Common Law Partner	32 (68.1)
Mother	2 (4.3)
Father	10 (21.3)
Both Parents	1 (2.1)
Mother in Law	1 (2.1)
Grandfather	1 (2.1)
Multiple Chronic Conditions of Older Person Supported	
3–5	17 (36.2)
6–8	15 (31.9)
9–12	12 (25.5)
13–15	3 (6.4)

*Because of rounding, at times, percentages add up to 99.9–100.1

**Table 6:** Description of Healthcare Providers (*n* = 42)

Category	*n* (%)
Sex	
Female	40 (95.2)
Male	2 (4.8)
Age	
18–44	17 (40.5)
45–64	25 (59.5)
Highest Education Achieved	
Graduated from Secondary School	1 (2.4)*
Diploma	7 (16.7)
Bachelor’s Degree	17 (40.5)
Master’s Degree	7 (16.7)
MD	4 (9.5)
Certificate	6 (14.3)
Professional Background	
Case Manager	1 (2.4)
Exercise Therapist	1 (2.4)
Nurse Practitioner	1 (2.4)
Personal Support Worker/Healthcare Aide	6 (14.3)
Pharmacist	1 (2.4)
Family Physician	4 (9.5)
Physiotherapist	3 (7.1)
Registered Dietician	1 (2.4)
Registered Nurse	12 (28.6)
Registered/Licensed Practical Nurse	7 (16.7)
Speech Language Pathologist	1 (2.4)
Social Worker	4 (9.5)
Years Practicing	
0–5 years	7 (16.7)
6–10 years	7 (16.7)
11–15 years	8 (19.0)
16–20 years	3 (7.1)
20+ years	17 (40.5)
Work Setting	
Primary Care	20 (47.6)
Home Care	19 (45.2)
Community Care	3 (7.1)

*Because of rounding, at times, percentages add up to 99.9–100.1

### Overview of the findings

The following sections include an introductory description of the context of the participants’ experience of managing MCC and six main themes. The experience of managing MCC was described as: (a) overwhelming, draining and complicated; (b) organizing pills and appointments, (c) being split into pieces, (d) doing what the doctor says, (e) relying on family and friends, and (f) having difficulty getting outside help. Within the presentation of each theme, similarities and differences between groups of participants are outlined, and illustrative quotes, which reference the participant group, province and participant number, are presented.

### Context

Participants spoke of the complexity of the MCC experience: multiple conditions that included not only physical, but also psychological conditions such as depression and anxiety; impacts of MCC on physical, psychological and social domains of health; managing many different medications for multiple conditions; seeing multiple health and social care providers; and health-related crises that lead to transitions to and from the acute care sector. Within this context, participants described how they had to accept the realities of gradual health decline associated with MCC and set realistic goals. Older adults explained, “*I’ve adapted to it as it’s happened…it’s been a gradual thing. You know, you get this problem and then you get this problem and then you have another problem and …I’ve just dealt with it*” (Older Person ON 111).

Caregivers described dealing with increasing needs and demands associated with MCC: “*If I was to say anything about being a caregiver, I’d say it’s tough, and as she gets worse, it gets tougher*” (Caregiver AB 216). Caregiver goals were focused on keeping the care recipient as well as possible, and in their homes: “*keep him stable, where he is now”* (Caregiver AB 206). Healthcare providers focused on maintaining “*quality of life*” and setting “*realistic goals*” for persons with MCC, as one participant described: *“So it’s really trying to find out what do they actually want that would improve their quality of life in view of their chronic conditions”* (Palliative Social Worker ON 03). It is within this duality, on one hand feeling hope to maintain function and continue to live at home, but on the other, accepting the reality of worsening illness and its implications, that the experience of MCC was described.

### The experience of managing multiple chronic conditions

#### Overwhelming, draining and complicated

All three groups described the experience of managing MCC as overwhelming, draining and complicated. Older persons described their experience as “*difficult,*” “*lousy*” and “*hard.*” Many outlined feeling “*frustrated*” that they have “*no strength,*” “*no energy*” and “*limited mobility,*” forcing them “*to give up a great deal*” of both the everyday activities such as walking across a room, as well as special activities, such as sports and hobbies. Several older people described feeling “*overwhelmed*” by their MCC, that it was “*hard to cope,*” they were “*tired of the pain,*” that they had “*no control over*” their health and there was “*nothing I can do to stop it.*” Conversely, a few older persons described their experience as “*good, usually,*” that limitations “*come and go,*” but that generally they were “*managing well*” and described themselves as “*lucky*”.

Caregivers described their experience of caring for an older person with MCC as “*overwhelming,*” “*frustrating,*” “*draining,*” “*exhausting,*” “*stressful,*” “*challenging,*” “*time-consuming*” and “*emotional.*” They described having to “*do it all*” and felt “*stretched,*” wanting, needing and sometimes not being able to “*be there all the time.*” A few caregivers who were providing care to a family member whose MCC were relatively stable reported “*managing well*” and saw themselves as “*fortunate*”.

Providers described caring for older persons with MCC and their caregivers as rewarding, yet also challenging. Some providers described the challenge of trying to address the multiplicity and complexity of physical and psychological issues: *“It can be very draining if they have a lot of conditions. If you care … it’s difficult not to think that you can help with everything and be all things to all”* (Physiotherapist ON 08). Providers expressed a sense of isolation and potential for burnout in caring for clients with MCC: “*I have to handle all these clients by myself and sometimes there’s struggle and burnout because you can feel frustrated*” (Primary Care Social Worker AB 302).

#### Organizing pills and appointments

All three participant groups discussed their experiences in organizing care when dealing with MCC. Older person and caregiver efforts centred primarily upon managing multiple medications and appointments. Examples included: obtaining medications though doctor’s appointments for prescription renewals and ordering refills from the pharmacy; organizing medications at home using dosette boxes; monitoring and managing side-effects; and managing changes to medications that frequently occurred after an acute care hospitalization. Caregivers described “*constantly looking at medication*” (Caregiver ON 224).

Older persons and caregivers described that much of their communication with providers, particularly family physicians and specialists, focused on medications. One participant described his appointments with his family doctor: “*I just tell her why I’m there and [the situation] …and then she just gives me another pill*” (Older Person ON 115). Medication discussions focused on changing medication dosages, adding new medications, and discontinuing others.

Several participants described major changes to medication after older persons with MCC were discharged from acute care hospitals. These changes were described as being abrupt; carried out by providers without input from older persons or caregivers regarding their preferences; and in some instances, were harmful*.* New medications brought challenges of new side-effects that required additional monitoring and action. Providers from all disciplines and work settings discussed the complexity of medication management for older persons with MCC. This complexity involved not only large numbers of medications, but also interactions between them: “*It’s also very overwhelming to them [older persons with MCC]. They’re on 15 different medications because everybody has been giving them all, but taken all together, it’s just too much*” (Family Physician, ON 16). Several providers discussed the challenges of evidenced-based care for this group, since many guidelines are based on evidence from younger clients and those with single rather than multiple conditions:

*“The complexity of it makes it a little difficult… My biggest challenge is trying to follow guidelines because when we have patients who have multiple conditions and it’s impossible to follow ten different clinical guidelines, otherwise you have them on 50 different medications, so it’s always that risk-benefit assessment … most of the clinical guidelines aren’t made looking at studies in the geriatric population”* (Pharmacist ON 05).

Caregivers in particular spoke about organizing appointments and accompanying the older person: “*I go with him to all the specialist appointments. I always go*” (Caregiver AB 206). Caregivers also described “*coordinating*,” “*making arrangements*,” “*scheduling*” and “*managing*” healthcare appointments, along with managing tasks surrounding visits, such as completing blood work, going for scans and tests, arranging transportation, and when needed, negotiating time off work.

#### Being split into pieces

Older persons with MCC and caregivers described challenges receiving services from multiple providers who focus on a single disease or single aspect of their health, and do not see them as a whole person. Care is often experienced as disjointed and lacking coordination. Similarly, providers described difficulties “*knowing the whole picture*” since each provider only has a “*piece.*” Some older persons explained that they don’t see themselves with a number of conditions, that it is all “*one.*” A participant described: *“[The MCC are] all interrelated anyway…they’re all me…it begins to sound like the rheumatoid arthritis ‘me’ and the diabetic ‘me’ but they’re all me”* (Older Person ON 113). This description spoke to an approach to care that was often centred on a single disease or condition, rather than holistic or person-centred in nature.

There was variation in the experiences of caregivers and older persons related to how care is coordinated among multiple providers. Many participants described that information from specialists was sent to their family doctor: “*I mean there’s communication, one way back to the family doctor*” (Caregiver AB 206). Others reported that the coordination process was mysterious, they didn’t know how it was done, but reported that it must be happening: “*I have no idea if things go to him [family doctor] or not. Now, when I see him, I do share what happened when I saw a specialist, but I really don’t know*” (Older Person ON 101). Some participants described the healthcare experience as piecemeal*:* “*I don’t see as much of a team approach. It’s very piecemeal”* (Caregiver ON 201).

Many providers discussed challenges in care coordination across the multiple specialists and providers and with family and patients. They described challenges in obtaining health information they needed to provide care in a timely way. This was particularly so for front-line home care providers: *“I have delayed or no, medical information… so, it can take several days for a phone call of information to come through to me”* (Physiotherapist ON 07). They explained they often rely on family members to act as communicators with the team: *“The patient or the patient’s family… often have a copy of the findings and they’ll show us. That is the only way”* (Registered Nurse Home Care ON 20).

Providers who worked in primary care teams described the ease of accessing information from other team members through the shared Electronic Medical Record (EMR): “*Every single discipline, they have access to other files from the different disciplines, which is helpful”* (Primary Care Social Worker AB 302). They also expressed a need for this information to be available to providers outside of their team (e.g., home care providers) in order for providers to have a holistic focus: *“The shared patient record is key for different agencies, it is invaluable in A) not duplicating and B) being aware of the full picture before you treat something in isolation”* (Registered Dietitian ON 06).

Not only was there a ‘split’ or division between care for different chronic disease conditions, there was also a separation between care for diseases and health promotion/disease prevention efforts. Older persons and caregivers rarely mentioned healthy eating, physical activity or other health promotion activities as the focus of physician interactions. However, many older adults and caregivers reported that they were engaging in health promoting activities such as “*watch what I eat,*” “*eat proper foods*” and “*walk*” to “*lose weight,*” as well as doing “*puzzles and games*” to keep the mind “*busy*” with cognitive exercise. Non-physician healthcare providers often mentioned health promotion activities as a key part of their care and described how they helped clients see the connections between health promotion activities and improved function.

#### Doing what the doctor says

There was a marked disparity between the experiences of older persons and caregivers compared with providers in relation to making decisions about managing MCC. Older persons and caregivers perceived that decision-making regarding their care was physician- or provider-directed. In contrast, providers perceived that they were using a shared decision-making and collaborative approach. One of the first steps of engaging older adults and caregivers in decision making involves actively listening to their concerns and experiences. A number of participants described instances of their voices and wishes not being heard by family physicians and specialists: “*We don’t get a chance to say anything to him because he’s ready to get out; he’s got so many clients, these specialists see as many patients as they can and you’re out of there. That’s disappointing*” (Caregiver ON 219).

When asked to describe the decision making process related to managing MCC, many older adults and caregivers reported “*obeying*” doctors, and that “*we do what the doctor tells us to do.*” They described barriers to being engaged in these decisions: “*Most doctors don’t get it…they hear what they want or what they think you’re supposed to say and they tell you what they want*” (Older Person AB 107). When asked if family doctors discussed goals of care, care planning for the future, or provided health information or connections to services, many participants reported they did not. In a few situations, participants described relationships with physicians where their concerns were heard and acted on:

“*The doctors will talk with me and listen to what I’m telling them and then we’ll try to work together on what we could do to keep him as comfortable as possible. I feel that I have a pretty good voice*” (Caregiver ON 222).

In stark contrast, providers from all disciplines and settings exhaustively described their interactions with older adults with MCC and family members as focusing on their priorities, collaborative, respecting choice, and fully engaging them in decision making related to care. They used phrases such as: “*My goals are to meet their goals,” “trying to let them steer their own boat,” “giving them choices” and “all my treatment decisions are made in collaboration with the patient.”* Providers described starting with patient and caregiver goals: “*My job is to recommend decisions to people or recommend treatment options. So, I make sure they understand their options as best as possible and I would start with ‘what’s your goals of care?’”* (Family Physician ON 11). Similarly, in home care, the focus was on clients and choice: “*It’s a collaborative effort, client being number one, what do they want? And then providing them with what options we have to offer*” (Registered Nurse Home Care AB 20).

#### Relying on family and friends

Participants in all three groups described the key role that family and friends play in providing informal support to persons with MCC. Older adults and providers described relying on these supports, while caregivers described the significant and often stressful roles they took on to help the older person with MCC to continue to live in their own home. Informal caregivers provided a very broad range of supports that addressed the complexity of MCC including physical care, provision of meals, housework, managing medications and their side effects, assisting with exercise, providing transportation, and accompanying older persons to medical and other appointments. Caregivers also provided emotional support, cognitive stimulation, social outings, and personalized care.

Informal support was most often provided by spouses and children: “w*ith the support of my husband and two daughters, we manage quite well*” (Older Person AB 104), and friends: “*I’ve got good support – friends…you gotta have that support system”* (Older Person AB 112). Participants also talked about other sources of support such as faith communities and online sources of support: “*I get a lot of support from friends and people I know on Facebook*” (Older Person ON 107).

Many participants from all three groups described the informal caregiver role of supporting individuals with MCC as one of excessive strain and burden. An older adult explained: “*She does everything. She’s got a tremendous load, a very tremendous load”* (Older Person AB 110). Caregivers described a sense of having to do everything without help: “*I think having to do everything, pretty well....some days it’s too much. I can’t handle all this and I don’t have any outside help*” (Caregiver ON 221). Caregivers recognized the seemingly unending nature of caregiving for persons with MCC as overwhelming: “*I feel like I’m floundering because I feel overwhelmed. I feel like there’s no end in sight”* (Caregiver AB 212).

Providers described the primacy of the role of informal caregivers in supporting care of older persons with MCC. They explained that having family members improves patient care and outcomes: “*if they have a family member nearby who’s willing and wanting to help, it makes a huge difference in terms of prognosis and the outcomes and that patient’s care”* (Pharmacist ON 05). Physicians expressed how the active involvement of caregivers facilitates more coordinated care: “*You always hope they have someone that’s there to help them out. Because there’s adults that don’t necessarily have someone that can just come to appointments or take them places or be involved in their healthcare and then it’s just a mess*” (Family Physician, ON 13). One provider beautifully illustrated this theme: *“I think their ability to come to those appointments, to attend education sessions, to be kind of a participant in that journey is key”* (Registered Dietitian ON 06).

#### Having difficulty getting outside help

All three groups of participants spoke of the difficulty in getting outside help to assist the older person and caregiver to manage the complexity of MCC in the home. Outside help included a wide range of health services such as home care, as well as support services such as caregiver support groups. Participants described five aspects of getting outside help: (a) the lack of awareness of available community health and social support services; (b) the difficulty in accessing services; (c) the inadequacy of services in meeting needs; (d) the delay in accessing or refusal of services, and (e) needing a ride.

#### The lack of awareness of available community health and social support services

Many caregivers and older persons reported being unaware of available community health and social support services: “*I don’t know of anything else. My granddaughter, she was saying that there’s a lot of stuff out there that we could have. I don’t know*” (Caregiver ON 217). Some reported hearing about services through providers or word of mouth. Many providers, in contrast, described awareness of support services, regularly discussing those services with clients, referring clients to “*a whole list of resources*” (Physiotherapist ON 07) and sharing contact information.

#### The difficulty in accessing services

Participants described the complexity of both accessing information about services as well as accessing the services themselves as this caregiver explained: “*What I have found is accessing information or services or knowing what resources are available is very complex. It is convoluted almost and there are so many people involved that you don’t know where to start*” (Caregiver AB 221). Some older adults and caregivers who had tried to access support described frustrations connecting with organizations, waitlists and being ineligible for services: “*I signed up for an exercise class and it took me a year before I got in*” (Older Person AB 101). A caregiver described her attempts to organize services for her husband but because his condition was not considered severe enough, they “*floundered*” and had to wait for years before he became eligible.

#### The inadequacy of services in meeting needs

All three participant groups spoke of the inadequacy of community health and support services in meeting needs. Most frequently, they spoke of the inadequate number of hours of homecare services to meet the complex and intense needs of older persons with MCC: “*For my father, he’s maxed out in terms of the number of hours; he’s got 90 hours a month but that’s only 3 hours a day and when he needs 24 hour coverage, that’s really hard*” (Caregiver ON 205); *They’re getting 2 hours a week and it’s really not enough”* (Family Physician ON 16); and “*There just isn’t enough community supports, there isn’t enough respite*” (Outreach Social Worker AB 01).

There was also variation in the perceived usefulness of home care with some participants reporting it as “*great*”, others as inconsistent, inconvenient, and some as no help at all. Participants spoke of the challenge of lack of consistency of home care staff: “*It’s a different person each time. That, to me just does not sit. If I have to show them what, or she has to tell them each time what to do, you might as well do it then*” (Caregiver AB 204). One respondent described that when her family member was deemed palliative she was able to receive much more support, with better coordination than when he was deemed chronically ill.

#### The delay in accessing or refusal of service

Many respondents indicated that they used no formal services at all, or that they used services in the past, but none currently: “*No, other than going to the doctors*” (Caregiver ON 208); “*No, no there’s nobody, really … No, I have nobody come*” (Older Person ON 106). Caregivers and older adults often described delaying or refusing services for a variety of reasons such as valuing their independence and personal preferences of the older person. Many caregivers stated that they were “*managing,*” so did not want the disruption that would come with acceptance of services: “*I’ve been offered respite, I’ve been offered help with light housekeeping and at the moment I can manage that so I really don’t want someone coming in among all this while I can still take care of it*” (Caregiver ON 222). Some caregivers spoke of the reluctance of the older person to participate in programs: “*Well, there’s things that we have access to that he doesn’t want to participate in, like programs outside the house. He was a part of that and he didn’t want to go anymore*” (Caregiver AB 214).

Providers explained that they struggled with situations where families with MCC clearly needed support services, yet refused these services. Reasons for refusal of services included pride, independence, a reluctance to have strangers in the home, and a fear that opening the door to services was indicative of an impending move to long term care. Providers struggled with how best to support families to accept services:

“*So getting them to open up to a wider scope of support … [the family says] ‘It’s alright, we’re handling this. ‘Well, they’re not because it’s getting worse and worse. Let us help you find the support; let us work together but, ‘Oh, no, we’ll do it. We’ll manage fine; you’re busy. You’ve got other people that you need to worry about other than us.’ And that seems to be the biggest struggle*” (Registered Practical Nurse Home Care ON 22)

#### Needing a ride

All three participant groups discussed the importance of being able to drive or have transportation to attend the many medical appointments associated with MCC and to participate in programs or services. An older person explained that “*part of my problem is that anywhere or anything I want to do, I have to get [my husband] to take me because I can’t drive now”* and that even though there was a program in her church she wanted to attend, *“I hate to burden him with that when he’s already got enough on his plate*” (Older Person ON 118). A provider explained: “*Others are relying on a son or a daughter to take them to their medical appointments and then others might be relying on public transport. In some cases that’s a barrier to them actually participating in a service they could potentially benefit from*” (Exercise Specialist Primary Care AB 07).

Participants identified the costs associated with public or private transportation for frequent appointments related to MCC as an important barrier to accessing needed care: “*Transportation is a big issue and finances because people just can’t afford it*” (Physiotherapist ON 08); and “[*patients say] ‘I can’t afford a taxi. My husband is not well, can’t drive me. My kids are busy. I don’t know how I’m going to get here.‘So really, getting people to their medical appointments can be challenging*” (Physiotherapist AB 18). Providers also recognized the barriers posed by living in rural areas: *“In the rural areas, transportation is an issue. It’s very costly for patients to get to appointments”* (Nurse Practitioner ON 09).

## Discussion

This is the first study to explore the experience of managing MCC from the perspective of older adults, caregivers and a variety of providers in diverse community settings, with interviews of 130 participants in two different Canadian provinces. Study findings shed new light on the experience of managing MCC and are considered from lens of the AHRQ MCCRN Conceptual Model of Complexity and Healthcare for Patients with MCC [8]. The two major components of this model, needs and services, are linked by the concept of complexity which is the gap between the person’s needs and the capacity of healthcare services to meet those needs [8].

This study provides a valuable understanding of the concept of need in the AHRQ MCCRN model [8]. A key study finding was the consistency of the description of the experience of MCC across the three participant groups as burdensome and challenging. Older adults with MCC described experiencing loss of functional ability, lack of energy, lack of control, and pain. They struggled with the emergence of each new condition and the steady worsening of these conditions. While other research has found that older adults with MCC experience a high symptom burden [11, 13], the current study found the experience to be much more of a burden than previous research suggests [12]. Caregivers in particular, expressed feeling overwhelmed and isolated by the complexity of caring for individuals with MCC and could see no end in sight. This sense of caregiver burden in dealing with MCC has not been well described previously [17]. In this study, as in the AHRQ MCCRN model, social support from family and others plays an important role in shaping the experience of need. Interestingly, providers also described feeling drained and isolated by the MCC experience. Previous research has identified that primary care clinicians struggle with uncertainties in providing guideline-based care for patients with MCC as guidelines are generally focused on single conditions [19, 21].

Study results indicate that health service provision for MCC (the second major concept in the AHRQ MCCRN model) is experienced as piecemeal and fragmented. The healthcare system is seen as focused on single disease conditions, not the complexity of multiple diseases or conditions. Older adults expressed frustration that they were not viewed as more than their individual diseases, that they were not considered holistically and that the approach to care was disjointed. Older adults, caregivers and providers all spoke of the challenges of communication and coordination of care, given the multiple providers involved with care for MCC. Previous research has identified that older adults with MCC desire to have individualized and coordinated care and continuity of care [14]. Similarly, one of the areas of difficulty specific to the management of multimorbidity found in a review of the perceptions of general practitioners involved the disorganization and fragmentation of healthcare [21].

The AHRQ MCCRN model includes the concept of access to community resources [8]. In this study, there were key challenges in linking older adults with MCC and their caregivers with appropriate resources and services to help them with care in the home. Not only is there a lack of awareness of and difficulty accessing such services, but participants spoke clearly about the inadequacy of these services. In particular, they spoke of the insufficient number of hours of home care services, the lack of continuity of care and the lack of respite services for caregivers. This study also found that older adults and caregivers are often resistant to accepting community support services for a variety of reasons such as wanting to remain independent, fear, and avoiding disruptions in the home. Future research is needed to explore this finding in more detail, as this understanding could lead to improved patient and caregiver linkages with relevant services.

Study findings revealed a stark contrast between the perspectives of patients and families compared to providers related to patient involvement in goal setting and care planning for MCC. This finding indicates a gap between needs and service provision (analogous to the needs/service gap in the AHRQ MCCRN model) [8], at the level of the patient/caregiver and provider relationship. Older adults and caregivers often felt that their views and opinions were not heard or valued. Further, they expressed that they were not active participants in setting goals and plans for care to deal with MCC. Similarly, a study of patients with chronic conditions in England found that none of the 23 older patients experienced explicit care planning discussions or received written documents outlining a negotiated care plan [14]. The study by Kuluski et al. found little alignment of goals across patient-caregiver and physician triads [18]. Providers in the current study, in contrast, felt that they were focused on patient goals and had a very collaborative approach to care of MCC. Previous research with primary care clinicians has identified barriers to shared decision-making and identified conflicts between clinician and patient goals of care related to MCC [19–21].

Much of the interview data supporting the disconnect between patient/caregiver and provider perspectives of communication and goal setting arose from patient/caregiver discussions about interactions with specialists and family physicians. There was little patient/caregiver data indicating that this disconnect also occurred with non-physician providers, indicating an area for more exploration. Our provider sample was largely composed of professionals from primary care and home care settings, including Registered Nurses, Licensed Practical Nurses, and health care aides, who spoke extensively of open communication and joint goal setting with patients/caregivers. While this sample fills an important gap in the current literature that focuses primarily on physician perspectives of managing MCC, including specialists and additional physicians and nurse practitioners in the sample might have been valuable for a broader understanding of communication and goal setting processes between patients, caregivers and providers.

Study findings lend support to the importance of contextual factors, such as the social determinants of health, as influencing the experience of MCC [8]. Participants related the challenges of having available transportation to attend the many appointments with doctors and other support services. Participants also expressed concern about the costs of transportation and other services. A study of rural adults with MCC acknowledged the influence of social determinants such as the cost of transportation, the availability of affordable fresh food, and the costs of medications as factors influencing the ability to manage MCC [23]. However this study was not specific to older adults. Overall, study findings indicate that there is a high level of complexity in the experience of managing MCC, that is, there is a large gap between the needs of older adults and family caregivers and the ability of the current health and social care system to meet those needs [8].

### Study strengths and limitations

A strength of this study was the large, diverse sample of older adults, caregivers and providers from multiple settings and provinces that contributed to “representative credibility” of the interpretive description approach [22]. While there was variation in the education and income levels of older adults and caregivers, there was little cultural diversity in the sample and this may influence the experience of MCC. Although a diverse range of healthcare providers was interviewed, the sample did not include physician specialists, a provider group that was often mentioned by participants, and only a few physicians. It was particularly challenging to recruit family physicians to the study, in spite of working through a number of primary care practices as recruitment sites. The lack of specialist participation and the limited participation by primary care physicians (*n* = 4) and nurse practitioners (*n* = 1) is a study limitation and future research should address this limitation. Future research with older adults and caregivers from diverse cultures could be important to understand their unique perspectives. More in-depth research to better understand the disconnect between the perspectives of patients/caregivers and a range of providers related to communication and goal-setting would be valuable.

### Implications for practice and policy

The common and intense experience of MCC as overwhelming, draining, and complicated suggests the need to assess how people are dealing with MCC and to use strategies to better support all three groups of participants. Healthcare providers can assess how older persons and family members are dealing with MCC and suggest services such as respite care or caregiver support services. The inclusion of a formal assessment of caregiver needs by home care providers is recommended, as well as interventions targeted to support these caregivers. For healthcare providers, opportunities to debrief about challenging situations with colleagues and to receive ongoing training on managing MCC would be valuable.

The theme of being split into pieces has implications for healthcare professionals and the larger system of care. Healthcare professionals can play important roles in using a more holistic lens to assess and support patients with MCC and their caregivers, beyond the single disease condition and beyond disease conditions alone. Use of a broad social determinants of health or a complexity lens would see providers discussing with patients and caregivers issues such as social support, transportation, and finances as key factors that contribute to management of MCC [24]. Further, discussing strategies to promote health of individuals, in spite of the presence of MCC, and preventing the worsening of existing conditions are key provider roles that could be emphasized. At a systems level, there is a need to explore new care processes and innovative approaches to MCC that use interprofessional models of care to address a diverse range of client and caregiver issues. Examples include the Guided Care approach and the IMPACT clinic [25–27]. There is also a need to explore improved electronic communication systems so that all partners in care, including homecare providers, can share information to improve coordination of care.

The striking contrast found between the perceptions of older adults and caregivers, compared to that of providers in relation to participation in goal setting and care planning, highlights the need for improved communication and collaboration between these groups. Providers are encouraged to use person and family centred approaches to care that involve listening to and acting on the voices of older persons and family members. Further, they are encouraged to actively involve older persons and families in identifying and setting care goals. New models of person-focused care should be explored. The Health TAPESTRY (Teams Advancing Patient Experience: Strengthening Quality) program, for example, includes intentional, proactive conversations about a person’s life and health goals and tailored interventions to meet those goals, where trained community volunteers work in partnership with interprofessional primary care teams [28]. Further evaluation of such models in primary care, home care and community-based settings is needed.

Programs of research that examine strategies designed to support care coordination and patient-focused, goal directed self management are being implemented in the real world and address the key transitions from hospital to home, commonly experienced by older adults with MCC [29, 30]. Naylor’s transitional care model involves advanced practice nurses who provide discharge planning and home follow-up. Coleman’s Care Transitions Intervention includes a transition coach who promotes continuity of care from hospital to home through a home visit and follow-up phone calls. Both of these models have resulted in lower hospitalization rates and reduced healthcare costs [29, 30].

The theme of having difficulty getting outside help has implications for providers and decision makers. Given the challenges experienced in accessing services, healthcare providers should pay increased attention to sharing information about available community health and support services with patients and families, and facilitating access to these services (e.g., making referrals, making connections with services on behalf of patients and families). A type of community navigator role is used by nurses in the Guided Care approach for older adults with MCC, for example [31]. Providers can play a key role in helping patients and families reach a point of accepting community services as important resources to help manage the complexity and demands of MCC in the home. At a system level, there is a clear need for increased availability of homecare services to support family caregivers with the intensive care demands in the home. Models of community and primary care that include community navigation roles to address the complexities of MCC should be evaluated [32]. Some recent innovations hold promise for increasing awareness of support services. In the European Union, EU-GENIE (European Generating Engagement in Networks Involvement) is an on-line service that creates maps of community support services based on an individual’s self-identified needs and interests [33]. Future initiatives should be focused on developing and evaluating similar technological online databases that can be used by the public and providers to obtain information about community support services.

## Conclusions

The experience of managing MCC in the community was one of high complexity, where there was a large gap between the needs of older adults and caregivers and the ability of health and social care systems to meet those needs [8]. Healthcare for MCC was experienced as piecemeal and fragmented, focused on single disease conditions, with little focus on the person and family as a whole. There was a disheartening disparity between the perceptions of patients and families compared to providers related to participation in goal setting and care planning for MCC. Participants conveyed a disconnect between the complex and intense needs for home and community support services to deal with MCC and their awareness of and access to such services. There was a complex intertwine between contextual issues such as the social determinants of health and health services in the experience of managing MCC that deserves more attention. These important findings provide a rich foundation for the design of processes of care to more optimally address the needs-service gap that is integral to the experience of managing MCC in the community.

## Abbreviations

AB: Alberta
ID: Interpretive description
MCC: Multiple chronic conditions
ON: Ontario
